# Supplementary material for: How scars shape the neural landscape: Key molecular mediators of TGF-β1’s anti-neuritogenic effects
Source: PLoS One. 2020 Nov 24;15(11):e0234950. doi: 10.1371/journal.pone.0234950 (PMC7685464; doi:10.1371/journal.pone.0234950)
Supplement: S1 Fig — (DOCX) [file pone.0234950.s001.docx]

**S1 Fig Expression of TGF-β Rl and Rll in ND7/23 cells**

SFM-primed, differentiated ND7/23 cells were seeded at a density of 1x10^4^ cells/ml in 4-well Lab-Tek ll Chamber Slides (Thermo Fisher Scientific,) and cultured for 4 days. Cells were fixed with 0.4 ml per well of 4% formaldehyde, (#28908, Thermo Fisher Scientific) for 15 minutes at room temperature. The media chambers were removed with a slide separator and the slides were rinsed with PBS + 5% dextrose and post-fixed with absolute acetone (Sigma Aldrich) at -20˚C for 10 minutes. After blocking with 5% normal horse serum (NHS) for 30 minutes, slides were incubated overnight at 4˚C with monoclonal anti-mouse TGF-βRI (1:10, D-1, Santa Cruz Biotechnology), monoclonal anti-mouse TGFβ RII (1:10, D-2, Santa Cruz Biotechnology) or buffered saline as a negative control. This was followed by application of secondary antibodies: goat anti-mouse IgG (H&L) AlexaFluor-488 (1:200, #A11001, Invitrogen Corporation, Waltham, MA). After rinsing, Vectashield Antifade Mounting Medium with DAPI (#H-1200, Vector Laboratories, Inc., Burlingame, CA) was applied to all slides, which were then cover-slipped.

***S1 Fig. Differentiated ND7/23 cells express two different TGFβ receptor subunits. A.*** *Photograph of ND7/23 cells cultured in SFM + rNGF for 4 days and reacted with antibodies against TGFβ RI (green fluorescence).* ***B.*** *Photograph of ND7/23 cells cultured in SFM + rNGF for 4 days and reacted with antibodies against TGFβ RII (green fluorescence). In both A and B, note extensive staining of the somata and neurites for both receptors. In both photographs, cell nuclei are DAPI-positive and emit blue fluorescence. Scale bar = 100 µm for both photographs.*
