# Supplementary material for: How scars shape the neural landscape: Key molecular mediators of TGF-β1’s anti-neuritogenic effects
Source: PLoS One. 2020 Nov 24;15(11):e0234950. doi: 10.1371/journal.pone.0234950 (PMC7685464; doi:10.1371/journal.pone.0234950)
Supplement: S2 Fig — (DOCX) [file pone.0234950.s002.docx]

**S2 Fig Effects of NGF on GSK-3β signaling in differentiated ND7/23 cells**

GSK-3β is well-known as a major signaling node that regulates neurogenesis, neuronal polarization and axonal growth and guidance [1, 2]. It is therefore reasonable to posit that its activity in ND7/23 cells may be regulated by the application of rNGF, which is used to induce their differentiation. The activity of GSK-3β is controlled by phosphorylation of Serine 9, which leads to its inactivation [3]. To confirm that NGF-induced morphological changes were mediated by inactivation of GSK-3β, 3x10^5^cells were seeded into 35mm dishes and incubated with 50ng/ml rNGF for 1day. The next day, cells were washed, pretreated with two different doses (0.2ug/ml, 0.6ug/ml) of anti-NGF (E-12; Santa Cruz Biotechnology) antibody for 1hr and then treated with either 0.5 or 50ng/ml of rNGF for another 1hr. A polyclonal rabbit anti-p-GSK-3β_Ser 9_ antibody (1:2000; Cell Signaling Technology) was used to detect GSK3β activity and t-GSK-3α/β (1:2000; Santa Cruz Biotechnology) was used as a loading control.

***S2 Fig. NGF regulates activation (i.e. phosphorylation) state of GSK-3β in ND7/23 cells.*** *Western blot illustrating changes in levels of p-GSK-3β and t-GSK-3α/β in cultured ND7/23 cells. Treatment with 50ng/ml rNGF increased levels of p-GSK-3β relative to those of t-GSK-3α/β. Treatment of cells with 50ng/ml rNGF together with one of two different concentration of anti-NGF antibody (0.2 µg/ml [lane 3] and 0.6 µg/ml [lane 4]) for 1hr prevented the upregulation of p-GSK-3β in a dose-dependent manner.*

**References**

1. Hur EM, Zhou FQ. GSK3 signalling in neural development. Nat Rev Neurosci. 2010;11(8):539-51. doi: 10.1038/nrn2870. PubMed PMID: 20648061; PubMed Central PMCID: PMCPMC3533361.

2. Zhou FQ, Zhou J, Dedhar S, Wu YH, Snider WD. NGF-induced axon growth is mediated by localized inactivation of GSK-3beta and functions of the microtubule plus end binding protein APC. Neuron. 2004;42(6):897-912. doi: 10.1016/j.neuron.2004.05.011. PubMed PMID: 15207235.

3. Sutherland C, Leighton IA, Cohen P. Inactivation of glycogen synthase kinase-3 beta by phosphorylation: new kinase connections in insulin and growth-factor signalling. The Biochemical journal. 1993;296 ( Pt 1):15-9. doi: 10.1042/bj2960015. PubMed PMID: 8250835; PubMed Central PMCID: PMCPMC1137648.
