# Supplementary material for: How scars shape the neural landscape: Key molecular mediators of TGF-β1’s anti-neuritogenic effects
Source: PLoS One. 2020 Nov 24;15(11):e0234950. doi: 10.1371/journal.pone.0234950 (PMC7685464; doi:10.1371/journal.pone.0234950)
Supplement: S3 Fig — (DOCX) [file pone.0234950.s003.docx]

**S3 Fig Effects of SIS3 and LiCl on Smad3 phosphorylation in ND/23 cells**

Passage 5, SFM-primed cells were seeded at a density of 3x10^5^cells per 35mm dish. They were incubated with 50ng/ml rNGF for 1day, washed and pretreated with 10ng/ml TGF-β1 with/without 5μM of the Smad3 inhibitor (SIS3, Sigma Aldrich) or 10 mM of the GSK-3β blocker LiCl (Sigma Aldrich) for 1hr. Finally, 100ng/ml rNGF was added for 1hr. We used Western blots to assay the expression of p-Smad3-Ser204 (polyclonal rabbit, 1:500, STJ90523, St John’s Laboratory Ltd.), p-Smad3-Ser423/425 (polyclonal rabbit, 1:500, STJ113511, St John’s Laboratory Ltd.) and t-Smad3 (polyclonal rabbit, 1:2000, #9523, Cell Signaling Technology). β-actin (monoclonal mouse, 1:10000, Santa Cruz) was used as a loading control.

***S3 Fig. Effect of NGF, TGF-β1, SIS3 and LiCl on 2 different phosphorylation sites on Smad3.*** *Western blot illustrating changes in levels of Smad3 phosphorylated at 2 different sites in cultured ND7/23 cells 1 hour after treatment with TGF-β1, LiCl or the Smad3 inhibitor SIS3. Note that TGF-β1 only upregulates p-Smad3-Ser204, and not p-Smad3-Ser423/425. Note also that SIS3 only affects levels of p-Smad3-Ser204, and not p-Smad3-Ser423/425. However, 2 different phosphorylated sites on Smad3 are completely inhibited by LiCl.*
