# Supplementary material for: How scars shape the neural landscape: Key molecular mediators of TGF-β1’s anti-neuritogenic effects
Source: PLoS One. 2020 Nov 24;15(11):e0234950. doi: 10.1371/journal.pone.0234950 (PMC7685464; doi:10.1371/journal.pone.0234950)
Supplement: S4 Fig — (DOCX) [file pone.0234950.s004.docx]

**S4 Fig Lack of dose-dependent effects of SIS3 on TGF-β1/p-GSK-3β signaling in ND/23 cells**

Passage 5, SFM-primed cells were seeded at a density of 3x10^5^cells per 35mm dish. They were incubated with 50ng/ml rNGF for 1day, washed and pretreated with 10ng/ml TGF-β1 with/without 1, 5, 10μM of the Specific Inhibitor of Smad3 (SIS3, Sigma Aldrich), or 10mM of the GSK-3β blocker, LiCl (Sigma Aldrich) for 1hr. Lastly, 100ng/ml rNGF was added for 1hr before harvesting to check for the expression of p-GSK-3 (polyclonal rabbit anti-p-GSK-3β_Ser 9_ antibody, 1:2000; Cell Signaling Technology).

***S4 Fig. Different concentrations of the Smad3 inhibitor SIS3 fail to impact levels of p-GSK-3β in ND7/23 cells.*** *Western blots illustrating changes in levels of p-GSK-3β in cultured ND7/23 cells 1 hour after treatment with TGF-β1, LiCl or different concentrations of the SMAD3 inhibitor SIS3 (1-10µM). In all cases, SIS3 failed to alter levels of p-GSK-3β relative to those attained with 10ng/ml TGF-β1 alone. This should be contrasted with the significant increase induced by administration of 10mM LiCl, which significantly increases levels of p-GSK-3β relative to those attained with 10ng/ml TGF-β1 alone.*
