# Supplementary material for: How scars shape the neural landscape: Key molecular mediators of TGF-β1’s anti-neuritogenic effects
Source: PLoS One. 2020 Nov 24;15(11):e0234950. doi: 10.1371/journal.pone.0234950 (PMC7685464; doi:10.1371/journal.pone.0234950)
Supplement: S1 Raw images — (PPTX) [file pone.0234950.s005.pptx]

## Slide 1
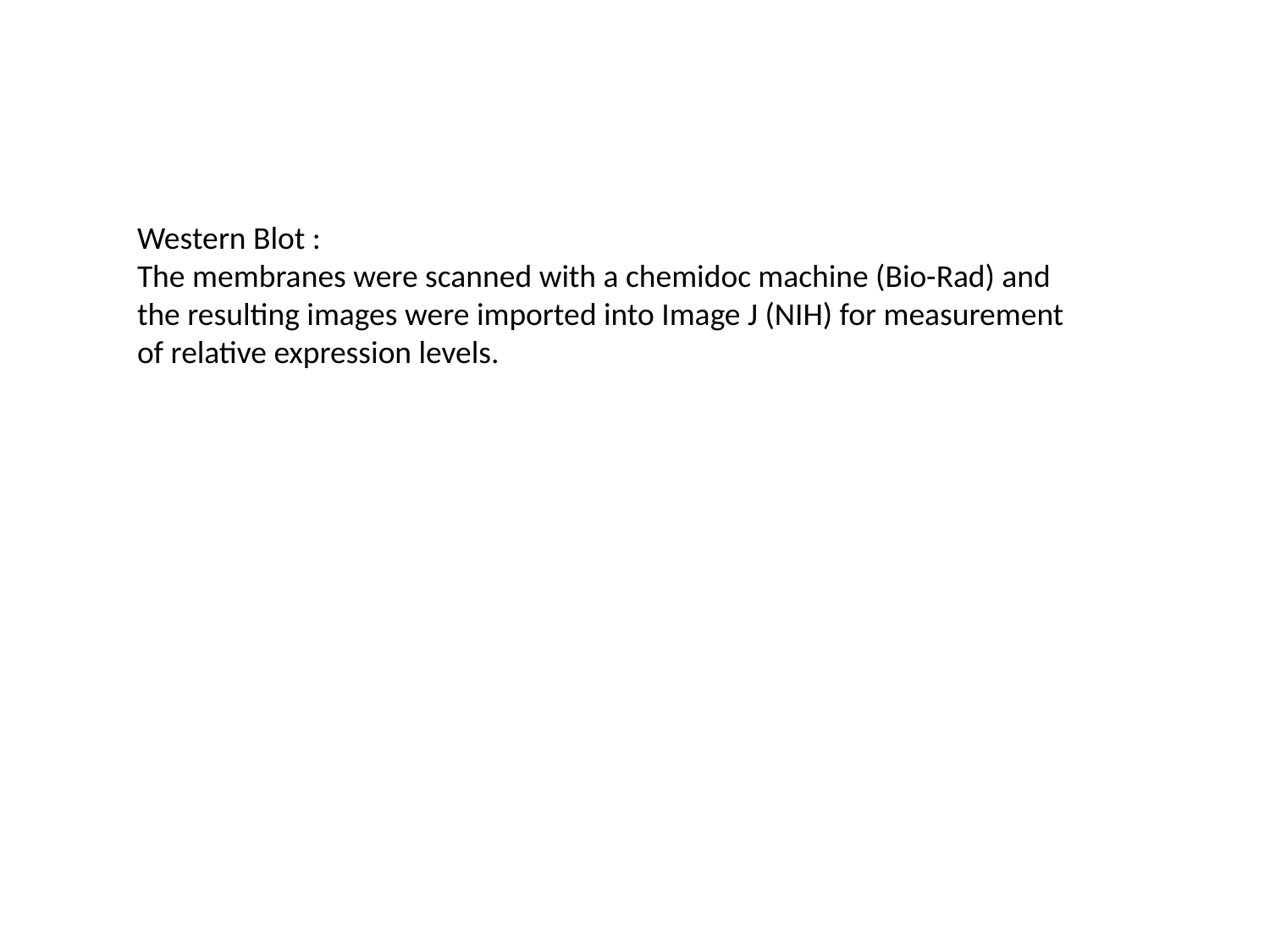

Western Blot :
The membranes were scanned with a chemidoc machine (Bio-Rad) and the resulting images were imported into Image J (NIH) for measurement of relative expression levels.

## Slide 2
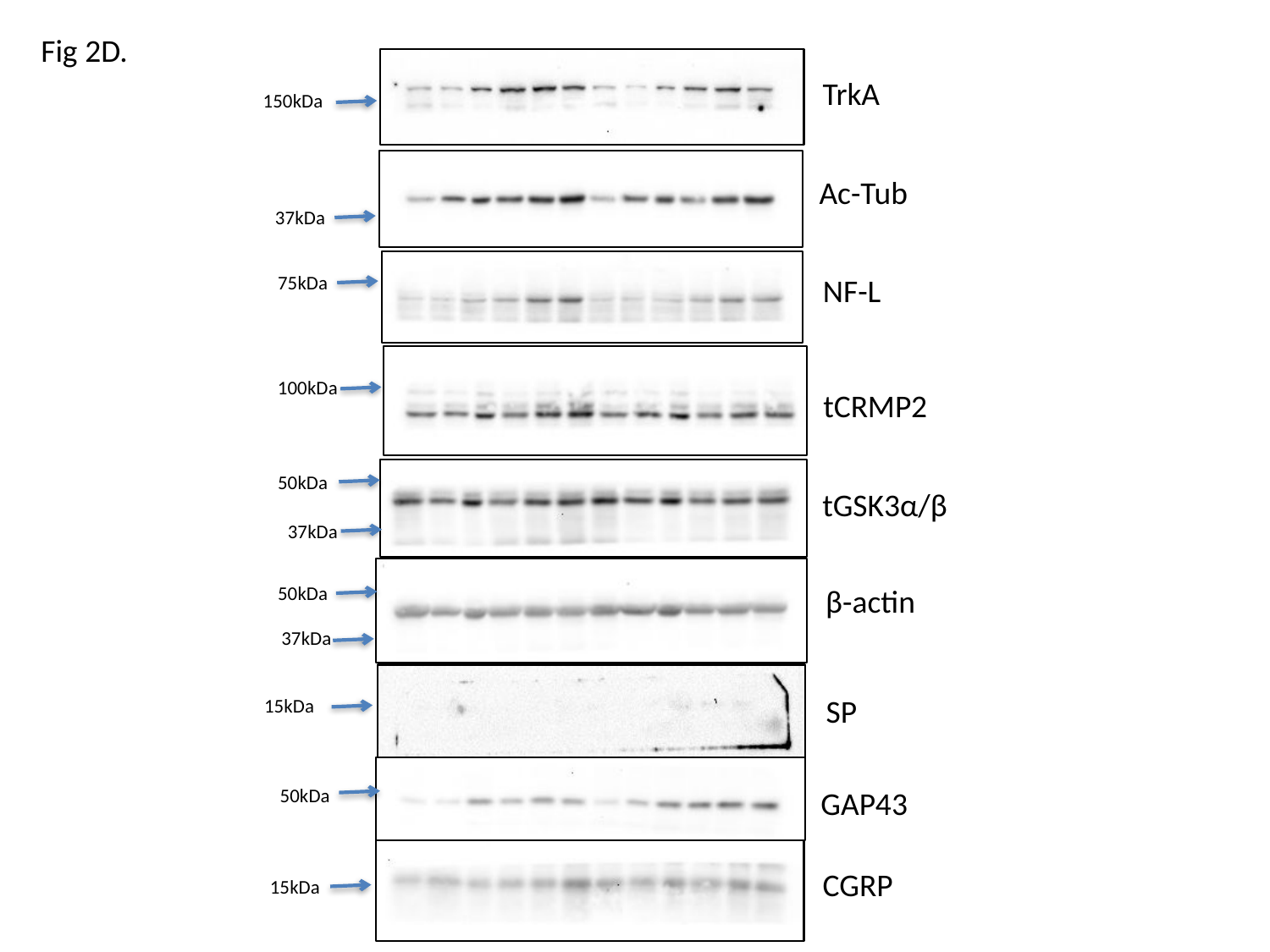

Fig 2D.
TrkA
150kDa
Ac-Tub
37kDa
75kDa
NF-L
100kDa
tCRMP2
50kDa
tGSK3α/β
37kDa
50kDa
β-actin
37kDa
SP
15kDa
50kDa
GAP43
CGRP
15kDa

## Slide 3
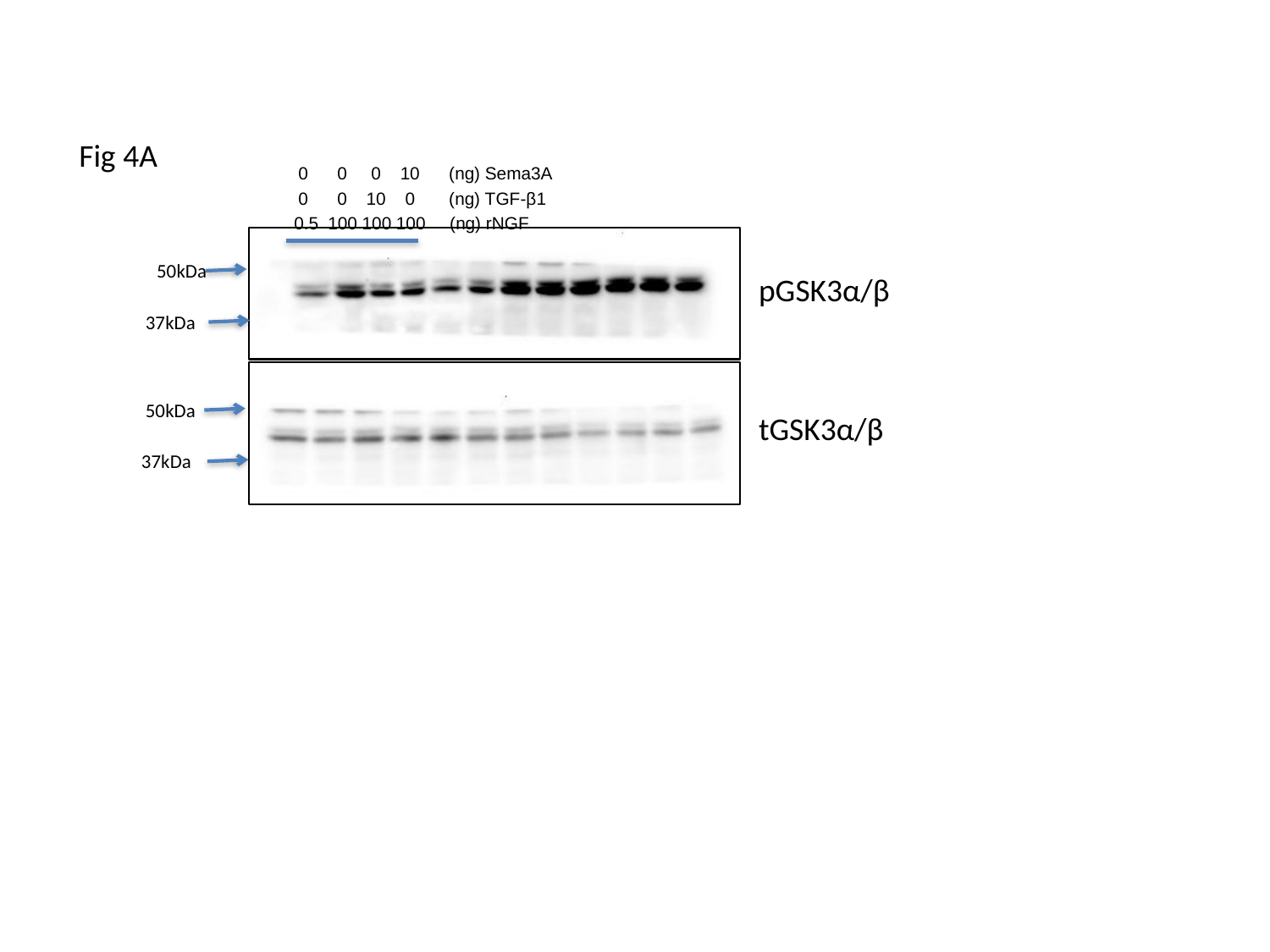

Fig 4A
0 0 0 10 (ng) Sema3A
0 0 10 0 (ng) TGF-β1
0.5 100 100 100 (ng) rNGF
50kDa
pGSK3α/β
37kDa
50kDa
tGSK3α/β
37kDa

## Slide 4
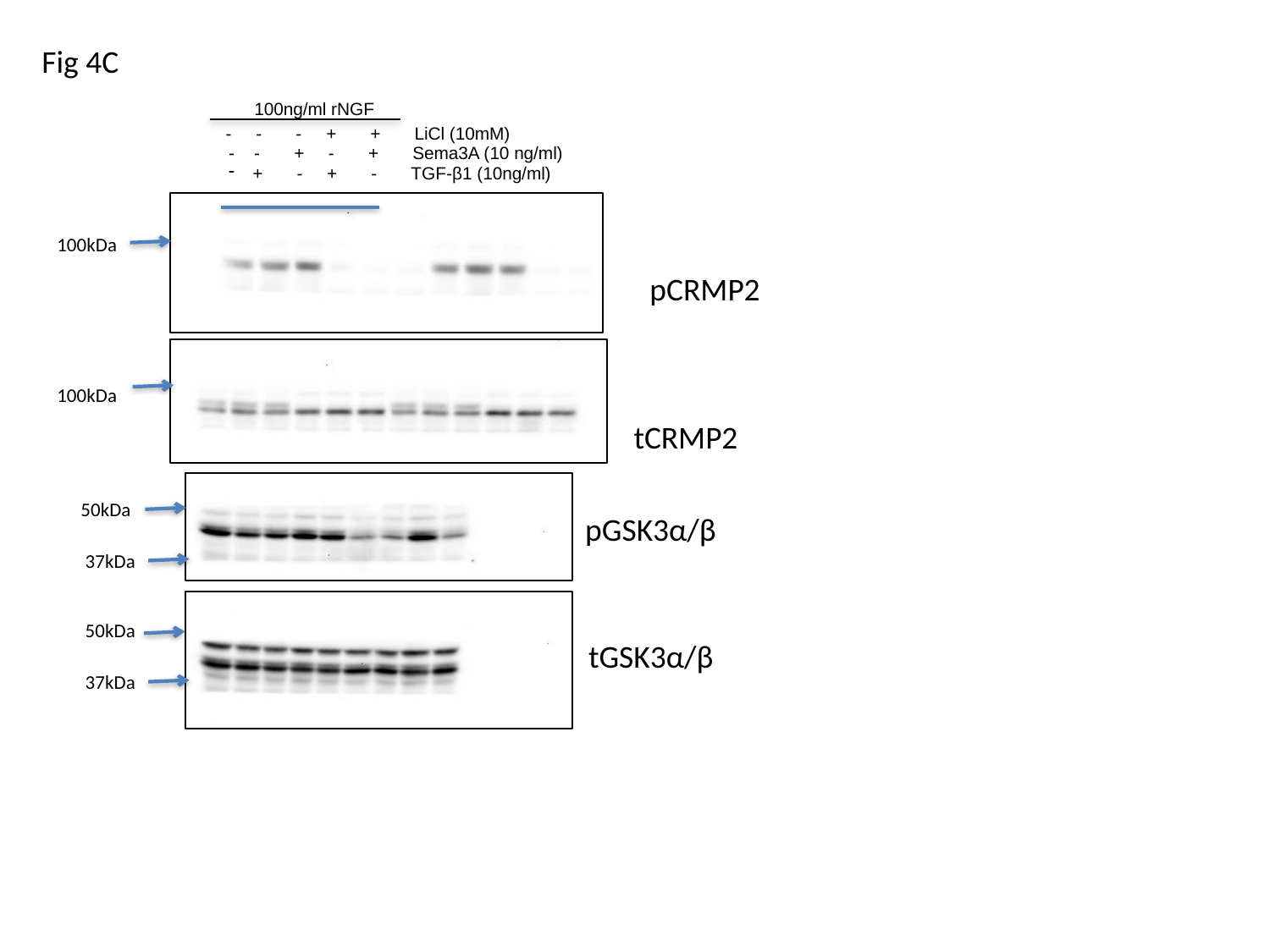

Fig 4C
100ng/ml rNGF
- - - + + LiCl (10mM)
- - + - + Sema3A (10 ng/ml)
+ - + - TGF-β1 (10ng/ml)
100kDa
pCRMP2
100kDa
tCRMP2
50kDa
pGSK3α/β
37kDa
50kDa
tGSK3α/β
37kDa

## Slide 5
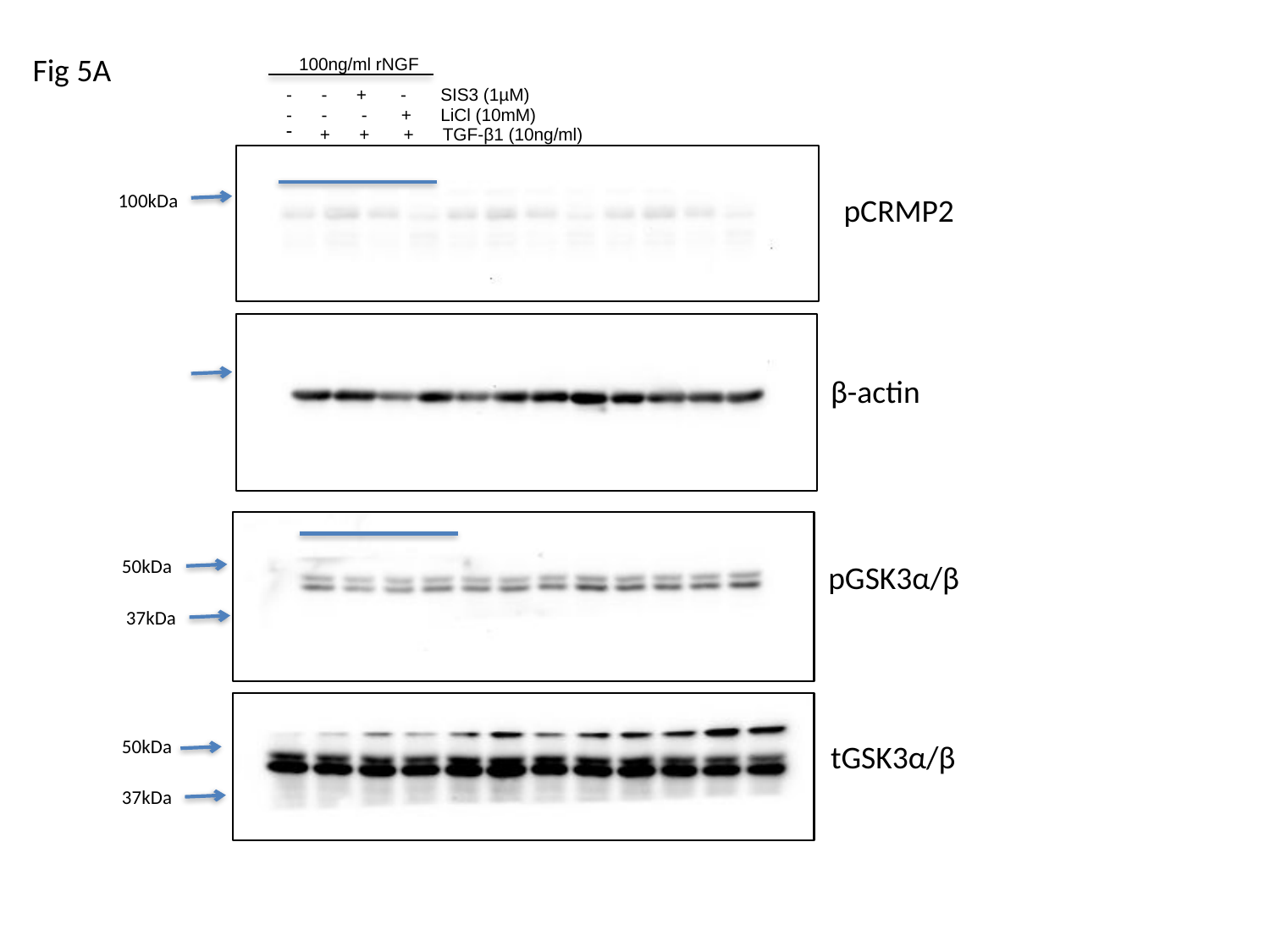

Fig 5A
100ng/ml rNGF
- - + - SIS3 (1µM)
- - - + LiCl (10mM)
 + + + TGF-β1 (10ng/ml)
100kDa
pCRMP2
β-actin
50kDa
pGSK3α/β
37kDa
50kDa
tGSK3α/β
37kDa
