# Supplementary material for: How scars shape the neural landscape: Key molecular mediators of TGF-β1’s anti-neuritogenic effects
Source: PLoS One. 2020 Nov 24;15(11):e0234950. doi: 10.1371/journal.pone.0234950 (PMC7685464; doi:10.1371/journal.pone.0234950)
Supplement: Attachment — Submitted filename: Response to reviewers.docx [file pone.0234950.s006.docx]

We thank the two reviewers for their time and attention, as well as constructive comments on our manuscript. We have carefully addressed each of the comments in the revised documents. Our point-by-point responses are indicated in blue text below. Revisions made to the manuscript are also indicated in blue. We hope that you will now find our revised manuscript suitable for publication in PLOS One and look forward to your evaluation.

**Reviewer #1**: The paper is technically complex and hence challenging to read. I found the message was lost in the details. It would be very helpful for the reader to have a "big picture" diagram as Figure 1.

*Response: we have added a new Figure1 that schematically summarizes the corneal wounding context in which we are working, and the specific signaling gaps we are attempting to fill. We have also modified the text of the introduction to more clearly motivate the present experiments, ending with the 2 specific questions we are asking about the role of GSK-3β and Smad signaling in mediating the anti-neuritogenic effects of TGF-β1 in sensory peripheral neurons such as those innervating the cornea.*

Also, the author states that the magnification was 10x or 20X. It is more likely that a 10X or 20X objective was used and that is in combination with 10X binoculars so the magnification would be 100X and 200X. Please check this.

*Response: since Reviewer 2 also brought up a related point, we now address both comments by providing scale bars on all photomicrographs presented in the Figures to this manuscript.*

**Reviewer #2**:  There are some minor points to be corrected:

1) Figure 1: Scale bar is missing in Figure 1A-C. Please insert one. Magnification indicators are not informative for the reader, please insert scale bars throughout the MS.

*Response: as noted above in response to Reviewer 1’s second comment, we now provide scale bars on all photomicrographs presented in the Figures to this manuscript.*

2) The authors write in the results’ section (Impact of TGF-β 1 on neurite outgrowth) “these cells were cultured with 50ng/ml rNGF for 1 day, the proportion of cells in Stage 1 decreased (two-tailed paired t-test for 0.5 versus 50 ng/ml rNGF conditions: t3=4.33, p=0.023), while the proportion of Stage 3 cells nearly doubled (two-tailed paired t-test: t3=3.58, p=0.037) – see pictures in Fig. 2E”. Figure 2E does not represent this statement. Please clarify.

*Response: it is difficult, given the neuronal density in these cultures to capture pictures of sufficient resolution to show morphology, while also capturing a sufficient number of cells to illustrate the quantitative data in A-D, which was based on hundreds of neurons. We removed the statement “– see pictures in Fig. 2E” from the text, but also added labels to panel E of the Figure to indicate what stages the cells pictured were in. The only picture in which Stage 3 cells are visible is the one of a culture treated with rNGF. The proportion of Stage1 and 2 cells captured photographically is greater in cultures treated with Sema3A or TGF-beta. This is reflective of our quantitative data.*

3) Please indicate the significant difference in Figure 2D

*Response: done*

4) What drug/treatment can block the effect of TGF-β1 in order to promote neurite outgrowth? Please discuss in detail. with specific details on molecular mechanisms.

*Response: we have added a relatively speculative paragraph to the end of the Discussion, answering this question.*
